# Crystal structure of a UDP-GlcNAc epimerase for surface polysaccharide biosynthesis in *Acinetobacter baumannii*

**DOI:** 10.1371/journal.pone.0191610

**Published:** 2018-01-19

**Authors:** Bhumika S. Shah, Heather E. Ashwood, Stephen J. Harrop, Daniel N. Farrugia, Ian T. Paulsen, Bridget C. Mabbutt

**Affiliations:** 1 Department of Molecular Sciences, Macquarie University, Sydney, Australia; 2 School of Physics, The University of New South Wales, Sydney, Australia; Monash University, AUSTRALIA

## Abstract

With new strains of *Acinetobacter baumannii* undergoing genomic analysis, it has been possible to define regions of genomic plasticity (RGPs), encoding specific adaptive elements. For a selected RGP from a community-derived isolate of *A*. *baumannii*, we outline sequences compatible with biosynthetic machinery of surface polysaccharides, specifically enzymes utilized in the dehydration and conversion of UDP-N-acetyl-D-glucosamine (UDP-D-GlcNAc). We have determined the crystal structure of one of these, the epimerase *Ab*-WbjB. This dehydratase belongs to the ‘extended’ short-chain dehydrogenase/reductase (SDR) family, related in fold to previously characterised enzymes CapE and FlaA1. Our 2.65Å resolution structure of *Ab*-WbjB shows a hexamer, organised into a trimer of chain pairs, with coenzyme NADP+ occupying each chain. Specific active-site interactions between each coenzyme and a lysine quaternary group of a neighbouring chain interconnect adjacent dimers, so stabilising the hexameric form. We show UDP-GlcNAc to be a specific substrate for *Ab*-WbjB, with binding evident by ITC (K_a_ = 0.23 μmol^-1^). The sequence of *Ab*-WbjB shows variation from the consensus active-site motifs of many SDR enzymes, demonstrating a likely catalytic role for a specific threonine sidechain (as an alternative to tyrosine) in the canonical active site chemistry of these epimerases.

## Introduction

*Acinetobacter baumannii* is a Gram-negative opportunistic pathogen responsible for a range of infections [1, 2]. It poses a serious clinical challenge due to its resistance to several classes of antimicrobial drugs [3, 4] and is today grouped as one of the highly multidrug resistant “ESKAPE” pathogens (*Enterococcus faecium*, *Staphylococcus aureus*, *Klebsiella pneumoniae*, *A*. *baumannii*, *Pseudomonas aeruginosa* and *Enterobacter species*) [5]. Wound infections caused by drug-resistant isolates of *A*. *baumannii* have been reported worldwide [6, 7], with outbreaks encountered recently in Australia [8] and the South Pacific [9]. *A*. *baumannii* is responsible for both community and hospital-acquired (nosocomial) infections that are difficult to control and treat [7]. Reports of community-acquired *A*. *baumannii* infections have intensified over the past decade [7, 8], particularly affecting those with a history of chronic alcoholism, cancer or chronic obstructive pulmonary disease [10]. Although community-acquired cases currently account for <10% of all *A*. *baumannii* infections, the associated mortality is considerable, ranging from 30–62% [6, 11].

Genome sequencing of various *A*. *baumannii* strains have revealed a marked genomic plasticity of this organism [2, 12]. A relatively small core genome can be defined, in conjunction with a large accessory genome [7, 8, 13]. Within individual genomes, regions of genomic plasticity (RGPs) are seen, encompassing genes offering niche advantage [8, 14]. Thus the pathogen’s ability to acquire foreign determinants through lateral gene transfer (LGT; or horizontal gene transfer) appears to provide the means for its rapid adaptation [15]. Furthermore, genomic and phenotypic analyses have described numerous virulence factors for *A*. *baumannii* [1, 3, 16]. Proposed factors include surface polysaccharides such as capsular polysaccharides (CPS), as well as proteinaceous components (porins, phospholipases, iron and zinc acquisition systems, protein secretion systems) and outer membrane vesicles [1, 3, 16].

Differentiation between CPS and O-antigenic components of lipopolysaccharide (LPS) and/or lipooligosaccharide (LOS) is challenging due to their similar structures and overlapping synthesis pathways. Consequently, there has been deliberation in the literature as to whether *A*. *baumannii* produces O-antigen and/or LOS and capsule. The specific ligase enzyme WaaL, which assembles LPS by covalently attaching lipid A-core oligosaccharide to O-antigen, is absent from *A*. *baumannii* genomes [17]. Whilst in certain *Acinetobacter* spp. one or two genes appear to encode proteins incorporating WaaL-like domains [17–20], these have subsequently been established to be enzymes of different function (e.g. PgIL enzyme [20] or for *O*-linked glycosylation [18]). It is thus currently presumed that *A*. *baumannii* produces a capsule and LOS (rather than O-antigen) to evade the innate immune response [17].

Across various *Acinetobacter* spp., gene clusters associated with synthesis of sugar components of CPS show great diversity [17]. This fosters structural heterogeneity of the CPS in terms of sugar composition and specific linkage chemistry [21]. During our own previous study of an *A*. *baumannii* community isolate (Australian strain D1279779), we identified a specific RGP encoding 11 proteins related to polysaccharide biosynthesis [8]. We describe here the characterisation and crystal structure for one of these, the enzyme *Ab-*WbjB, proposed to initiate conversion of UDP-GlcNAc to UDP-FucNAc. This specific protein was successfully produced as a result of high-throughput structural genomics screening across a wider pool of uncharacterised genes contained within *A*. *baumannii* RGPs. Like other dehydratases central to the biosynthesis of deoxy sugars [22], *Ab-*WbjB is organised as a short-chain dehydrogenase/reductase (SDR) fold. By detailing the catalytic site and oligomeric properties of this distinct dehydratase, we are here able to clarify molecular mechanisms impacting the biosynthesis of polysaccharides in *A*. *baumannii*.

## Materials and methods

### Bioinformatics analysis of island RGP01

Reciprocal BLASTP searches [23] were carried out on ten *A*. *baumannii* genomes against island elements of RGP01 from *A*. *baumannii* Dl279779 (locus tags ABD1_530 –ABD1_630). PSI-BLAST searches were then performed with default parameters [24] (see Table 1).

**Table 1:** Proteins encoded within RGP01 island of *A*. *baumannii* Dl279779.

locus	NCBI gene annotation	#amino acids	PSI-BLAST homologs^a^	% identity (E value)
**ABD1_00530**	UDP-glucose 4-epimerase	346	UDP-glucose 4-epimerase (*Alishewanella agri* BL06)	70 (3e-178)
			WbpP (*Pseudomonas aeruginosa*)	69 (2e-171)
			WbqB (*Escherichia coli*)	63 (7e-159)
**ABD1_00540**	polysaccharide biosynthesis protein	413	O-antigen translocase (*Xenorhabdus bovienii* SS-2004)	30 (2e-07)
			Wzx, ORF_11 (*Pseudomonas aeruginosa)*	22 (1e-06)
**ABD1_00550**	hypothetical protein	311	putative membrane protein (*Vibrio cholerae* HENC-03)	29 (2e-07)
			hypothetical protein (*Francisella cf*. *novicida* Fx1)	32 (3e-05)
**ABD1_00560**	hypothetical protein	392	WepQ (*Cronobacter sakazakii)*	35 (7e-39)
			group 1 glycosyl transferase (*Thauera sp*. MZ1T)	26 (3e-35)
**ABD1_00570**	glycosyl transferase	381	Mannosyl transferase (*Psychroflexus torquis)*	34 (6e-13)
			WfbF protein (*Pseudomonas fuscovaginae)*	26 (5e-9)
			Spore coat protein SA (*Bacillus thuringiensis)*	34 (1e-6)
**ABD1_** **00580**	UDP-N-	344	FnlA (*E*. *coli* STEC_EH250)	85 (0.0)
	acetylglucosamine 4,6-		WbjB (*Pseudomonas aeruginosa)*	81 (0.0)
	dehydratase, WbjB		Cap8E (*Staphylococcus aureus)*	70 (0.0)
**ABD1_** **00590**	WbjC	369	FnlB (*Escherichia coli)*	57 (3e-151)
			WbjC (*Pseudomonas aeruginosa)*	58 (2e-158)
			Cap8F (*Staphylococcus aureus)*	43 (0.0)
**ABD1_** **00600**	UDP-N-	376	WbjD (*Pseudomonas aeruginosa)*	81 (0.0)
	acetylglucosamine 2-		FnlC (*Escherichia coli)*	72 (0.0)
	epimerase		Cap8G (*Staphylococcus aureus)*	54 (0.0)
**ABD1_** **00610**	probable	310	WbjE (Pseudomonas aeruginosa)	42 (3e-88)
	glycosyltransferase		CapL (*Bacillus cereus*)	30 (1e-29)
			Cap8L (*Staphylococcus aureus*)	24 (1e-17)
ABD1_ 00620	UDP-glucose 4-	311	WbjF (*Pseudomonas aeruginosa)*	44 (2e-76)
	epimerase		probable UDP-galactose 4-epimerase (*Vibrio cholerae)*	46 (3e-83)
**ABD1_** **00630**	undecaprenyl-	336	WbpL (*Pseudomonas aeruginosa*)	50 (3e-99)
	phosphate N-acetylglucosaminyl 1-phosphate transferase		WbpL (*Pseudomonas sp*. R81)	49 (2e-103)

^**a**^ Searches conducted on non-redundant database, August 2017.

### Isolation of recombinant *Ab-*WbjB

The ABD1_580 gene was amplified by PCR using genomic DNA isolated from *A*. *baumannii* strain D1279779. The gene was sub-cloned into vector pET15b with *BamH1* and *Nde1* restriction sites to encode *Ab-*WbjB protein product with an N-terminal His_6_-tag. Recombinant expression of Se-Met labelled *Ab-*WbjB and its purification on Ni-affinity media were carried out by conventional laboratory protocols [25].

Purity of recombinant product (41 kDa) was verified using SDS-PAGE [26] (see S1 Fig). Oligomerisation state was analysed by size-exclusion chromatography (SEC) using Superdex 200 matrix (10/300 GL, GE Healthcare) pre-equilibrated in Buffer A (50 mM HEPES buffer, pH 7.5, 200 mM NaCl, and 5% v/v glycerol) operating at 0.5 mL/min. Under reducing conditions, chromatography utilised Buffer A supplemented with 50 mM *tris* (2-carboxyethyl) phosphine (TCEP).

### Crystallization and data collection

*Ab-*WbjB protein aliquots (13 mg/mL) were subjected to sparse-matrix crystal screen (96 conditions, MCSG-1, Microlytic North America) in sitting-drop vapour diffusion format with a Phoenix robot. Several crystals grew in 0.2 M NaCl, 0.1 M HEPES buffer (pH 7.5) and 25% v/v PEG3350, but diffracted at very low resolution (>7.5 Å). After extensive optimization (24-well grid, hanging-drop format) across varying pH and protein: precipitant ratios (1:1, 1:2 and 2:3), best diffracting crystals (<3.0 Å) were obtained at pH 5.6 and 1:1 mixtures.

Prior to flash cooling for data collection, crystals were soaked (10 min) in mother liquor supplemented with glycerol (20% v/v). X-ray data were recorded on beam lines MX1 and MX2 at the Australian Synchrotron (Melbourne) using *Blu-Ice* software [27]. Reflections were measured on an ADSC Quantum 210r detector (Poway, USA) at a wavelength of 0.9537 Å (13000.5 eV). Several data sets were collected due to radiation damage. Reflections were indexed, integrated and scaled with XDS [28]. Two datasets with lowest B factor values, diffracting at 2.70 Å (data set 1) and 2.64 Å (data set 2), were chosen for structure solution. Data set 2 consisted of a combination of 5 different wedges taken from the same crystal (120 frames each). Data with B-factors < -10 were removed from the latter data set. Full data collection and structure solution statistics are given in Table 2.

**Table 2:** Diffraction data statistics for *Ab-*WbjB ^a^.

	Data set 1	Data set 2
**Beamline (Australian Synchrotron)**	MX1	MX2
**Wavelength (Å)**	0.9537	0.9538
**Space group**	P2_1_2_1_2_1_	P2_1_2_1_2_1_
**a, b, c (Å)**	107.7, 114.2, 215.0	107.6, 114.3, 214.6
**α, β, γ (°)**	90, 90, 90	90, 90, 90
**Resolution (Å)**	19.97–2.70 (2.85–2.70)	24.83–2.64 (2.78–2.64)
**Unique reflections**	73156 (10507)	77374 (10206)
**Completeness (%)**	99.6 (99.2)	98.9 (94.6)
**Multiplicity**	15 (14.8)	6.9 (6.7)
**Mean *I/σ(I)***	13.4 (1.6)	13.4 (2.9)
**R_*merge*_ (%)**	0.264 (1.991)	0.103 (0.689)
**CC_1/2_**	0.995 (0.554)	0.996 (0.725)
**Anomalous completeness**	99.6 (98.7)	95.3 (90.4)
**Anomalous multiplicity**	7.8 (7.6)	3.6 (3.5)
**CC_anom_**	0.220 (0.016)	0.322 (0.022)

^a^ Values in parentheses denote the highest resolution shell.

### X-ray structure determination

A preliminary solution was obtained by molecular replacement (MR) with PHASER MR [29] from CCP4 [30] using the structure of *Helicobacter pylori* FlaA1 (PDB ID 2gn4; 39% sequence identity) [31]. To overcome relatively poor electron density (a result of low resolution, radiation damage and low sequence identity to model), single-wavelength anomalous diffraction (SAD) was utilised. A combination of both methods was used to generate the solution for data set 1, as the anomalous signal of crystals although relatively weak (see Table 2), was sufficient to bootstrap from a preliminary partial solution. This was carried out with *PHASER EP* [29] and identified 83 Se sites in each asymmetric unit (asu). Electron density maps were improved in *PARROT* [32] with advantage of a six-fold non-crystallographic local symmetry (NCS). Auto-building was initiated from both data sets 1 and 2 in *BUCCANEER* [33, 34] to further improve resolution of the structure to 2.65 Å. The final model was obtained after rounds of refinement in PHENIX [35] and manual model building in Coot [36]. The torsion NCS restraints were applied for better refinement of the structure.

The refinement statistics for the structure solved at 2.65 Å resolution are summarized in Table 3. The crystalline packing shows *Ab-*WbjB to be a homomeric hexamer, organized as a trimer of dimers (chains AD, BF and CE). Our final model contains coenzyme NADP+ bound to each of the six chains, as well as 160 water molecules per crystallographic asu. The relatively better stereochemical quality of Chains A and B over the remaining four chains is evident from their lower B factor values (see Table 3). Electron density is missing for the following residues: Asp246-Pro248 (Chain D only), Ser250-Glu258 (Chains B, E and F), Arg287-Glu306 (all Chains) and C-terminal 11 residues, Leu334-Ala344 (except Chain B). The overall stereochemical quality of the final model was assessed using MolProbity [37] and the ADIT validation server (http://deposit.pdb.org/validate/). Coordinates have been deposited within the Protein Data Bank as PDB file 4j2o. Structural homologs for *Ab-*WbjB were identified with DALI searches [38], last accessed in August 2017. All molecular views presented here were prepared using PyMOL [39].

**Table 3:** Refinement statistics for *Ab-*WbjB (PDB 4j2o).

Refinement	
**Resolution (Å)**	24.86–2.65
**σ cutoff**	*F* > 0.63σ(*F*)
**No. of reflections**	144937
***R*_cryst_*/ R*_free_ (%)**	16.0 / 20.1
**No. of atoms**	15096
**protein/no. of residues**	14937
**solvent**	159
**TLS groups**	17
**R.m.s deviations from standard values**	
**bond lengths (Å)**	0.01
**bond angles (°)**	1.301
**Average *B* factor (Å^2^)**	
**Chain A (main-chain/side-chain)**	58.8/66.4
**Chain B (main-chain/side-chain)**	55.8/64.8
**Chain C (main-chain/side-chain)**	68.5/76.1
**Chain D (main-chain/side-chain)**	70.9/77.5
**Chain E (main-chain/side-chain)**	80.1/85.5
**Chain F (main-chain/side-chain)**	84.0/88.4
**solvent**	62.8
**Ramachandran plot (%)**	
**favoured regions**	97.6
**allowed regions**	2.1
**outliers**	0.3
**PDB accession**	4j2o

### Site-directed mutagenesis

To generate mutants M134A-WbjB and M134Y-WbjB, isolated pET15b plasmids harbouring *fnlA* gene were subjected to site-directed mutagenesis with a commercial kit (QuikChange, Stratagene) and mutagenic primers (see S1 Table). After mutagenesis was confirmed via DNA sequencing, mutants were transformed into *E*. *coli* BL21 (DE3) pLysS expression host for protein expression and purification (as above).

### Differential scanning fluorimetry (DSF)

Fluorescence-based thermal melt analysis, DSF, was utilized to identify ligands which enhanced the stability of *Ab-*WbjB in solution [40]. Pure protein samples (10 mg/mL) were mixed with SYPRO Orange dye (200 x concentration; Invitrogen) and diluted (1:10) into Buffer A. Aliquots (10 μL) were placed into 96-well plates with queried conditions (10 μL) and temperature gradient applied (25–95°C, at 1°C/min). DSF responses were viewed in the MxPro q-PCR program (Stratagene). Each condition was repeated in triplicate, and derivative curves generated to determine the midpoint protein melting temperature (T_m_). Conditions resulting in a change of T_m_ >2°C were deemed significant. Potential *Ab-*WbjB substrates were investigated following screening of 96 cocktail combinations (5% v/v) from a commercial screen (Silver bullets, Hampton Research) [41].

### Isothermal titration calorimetry (ITC)

Protein samples in HEPES buffer (50 mM, pH 7.5), 200 mM NaCl and 0.5 mM TCEP were adjusted to 100 μM, and NADP+ added to 1 mM. ITC measurements were carried out at 25°C using a MicroCal Auto-iTC200 instrument (GE-Healthcare). Aliquots (2 μL) of potential ligands (1 mM) were injected into protein samples until binding appeared saturated (18 cycles, at 150 s intervals). Titrations of ligand buffer-only samples were performed to provide baseline readings. The corrected heat change was fitted in Origin 7 software (Microcal Inc., U.S.A) to obtain binding affinity constants (K_a_) and enthalpy of binding (ΔH).

## Results and discussion

### *Ab-*WbjB encoded within the polysaccharide biosynthesis RGP

**Fig 1:**
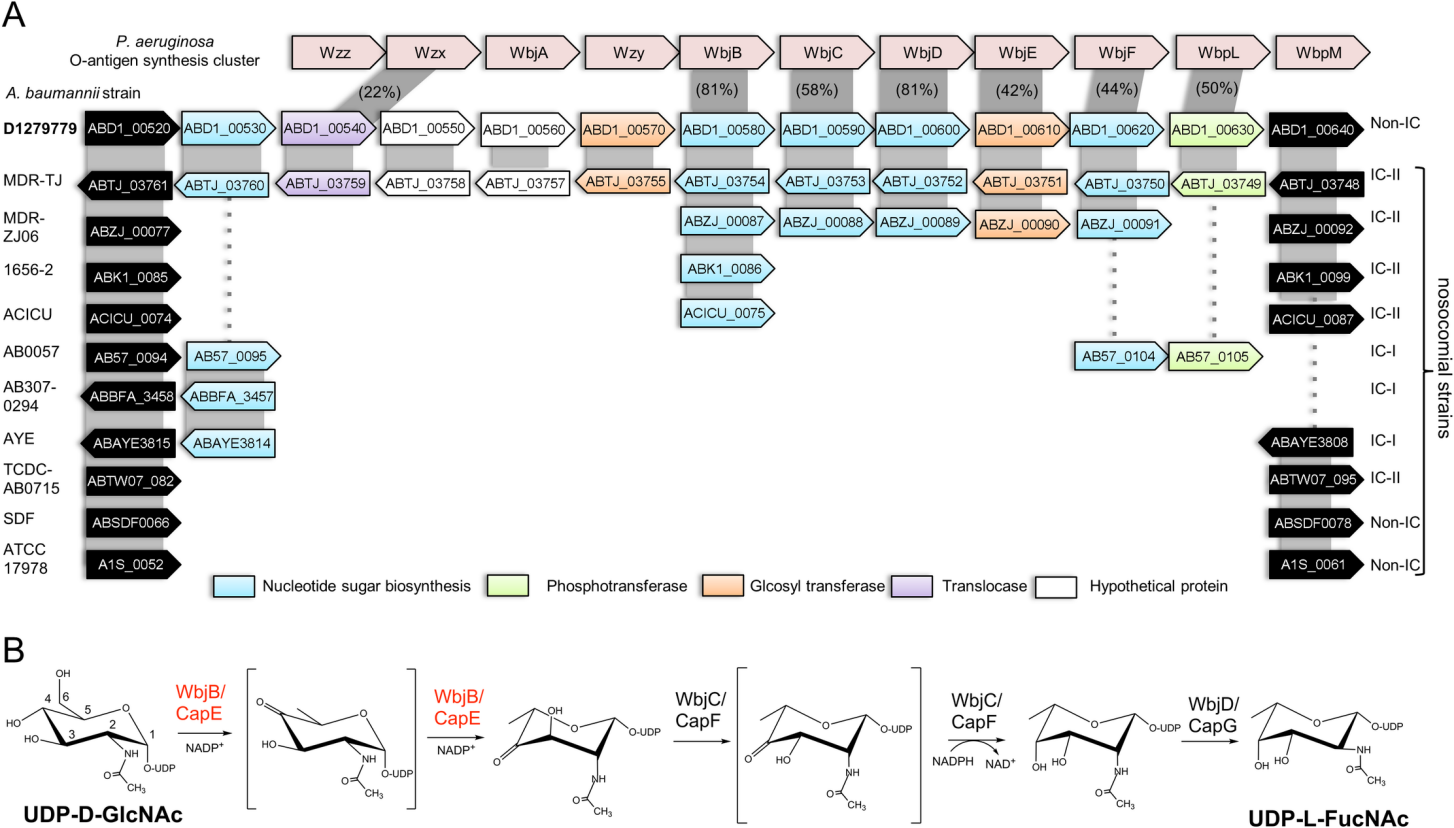
Components of polysaccharide biosynthesis cluster RGP01. (A) Gene conservation between the RGP01 island in *A*. *baumannii* D1279779 and related components of selected *A*. *baumannii* strains (clonal lineage indicated, right). For each component gene, locus tag and transcription direction are indicated. Colour is used to categorize functional annotations (from NCBI), with flanking chromosomal regions in black. Alignment with elements of the O-antigen biosynthesis cluster in *P*. *aeruginosa* is incorporated at top (red), with % sequence identity indicated for each orthologous pair. (B) Five-step biosynthesis of UDP-L-FucNAc by enzymes WbjB/CapE, WbjC/CapF, and WbjD/CapG as demonstrated in *P*. *aeruginosa* and *S*. *aureus* [43, 44].

Previous analysis of the genome of *A*. *baumannii* D1279779, a community isolate, has recorded 24 distinct regions of genomic plasticity [8]. Remarkably, none of these encode known antibiotic resistance elements common in *A*. *baumannii* nosocomial isolates [2]. Fig 1 outlines one such cluster of interest, described as RGP01, containing elements associated with oligosaccharide biosynthesis [8]. Whilst the same island also recurs in its entirety in the clinically-sourced *A*. *baumannii* strain MDR-TJ [42], it is absent from other *A*. *baumannii* genomes analysed to date. A few elements of the RGP01 island are also found in the nosocomial *A*. *baumannii* strain MDR-ZJ06, however, suggesting an origin via LGT. Notably, the specific gene component ABD1_00580, annotated as a UDP-N-acetyl-glucosamine 4,6-dehydratase, is found in orthologous forms across several nosocomial strains of the IC-II lineage of *A*. *baumannii*.

For seven component genes of RGP01, sequence matches can be found to distinct bacterial epimerase and related polysaccharide synthesis enzymes (Table 1). Recurring within these homologs is a specific O–antigen biosynthesis locus defined in *P*. *aeruginosa* O11 [45]: overall, 6 of 11 RGP01 genes share >40% identity with this cluster. Fig 1 includes a schematic relating the two clusters, most similar for those genes attributed to nucleotide sugar biosynthesis. Immediately obvious are enzymes WbjB, WbjC and WbjD, previously demonstrated in *P*. *aeruginosa* to convert UDP-N-acetyl-D-glucosamine (UDP-D-GlcNAc) to UDP-N-acetyl-L-fucosamine (UDP-L-FucNAc) [44]. The same suite of enzymes has also been described in Gram-negative bacteria such as *E*. *coli*, *Streptococcus pneumoniae* (named FnlA, FnlB, FnlC), and *S*. *aureus* (CapE, CapF, CapG).

*A*. *baumannii* genes ABD1_580, ABD1_590 and ABD1_600 are therefore attributed to encode the corresponding enzymes, *Ab-*WbjB, *Ab-*WbjC and *Ab-*WbjD, respectively. Based on the high sequence identity to their *P*. *aeruginosa* and *S*. *aureus* homologs, we propose these enzymes to be responsible for the conversion of UDP-D-GlcNAc to UDP-L-FucNAc [43] via the five-step reaction cascade characterized for these relatives (Fig 1B). The enzyme WbjB (like CapE) is bifunctional, first catalyzing the 4,6-dehydration of UDP-D-GlcNAc and, subsequently, a C5-epimerization step. Next, WbjC (like CapF) catalyzes a C3-epimerization step, followed by C4-reduction of the intermediate keto-sugar. The third associated enzyme, WbjD (or CapG), yields the product UDP-L-FucNAc via C2-epimerization.

### Three-dimensional structure of *Ab-*WbjB

**Fig 2:**
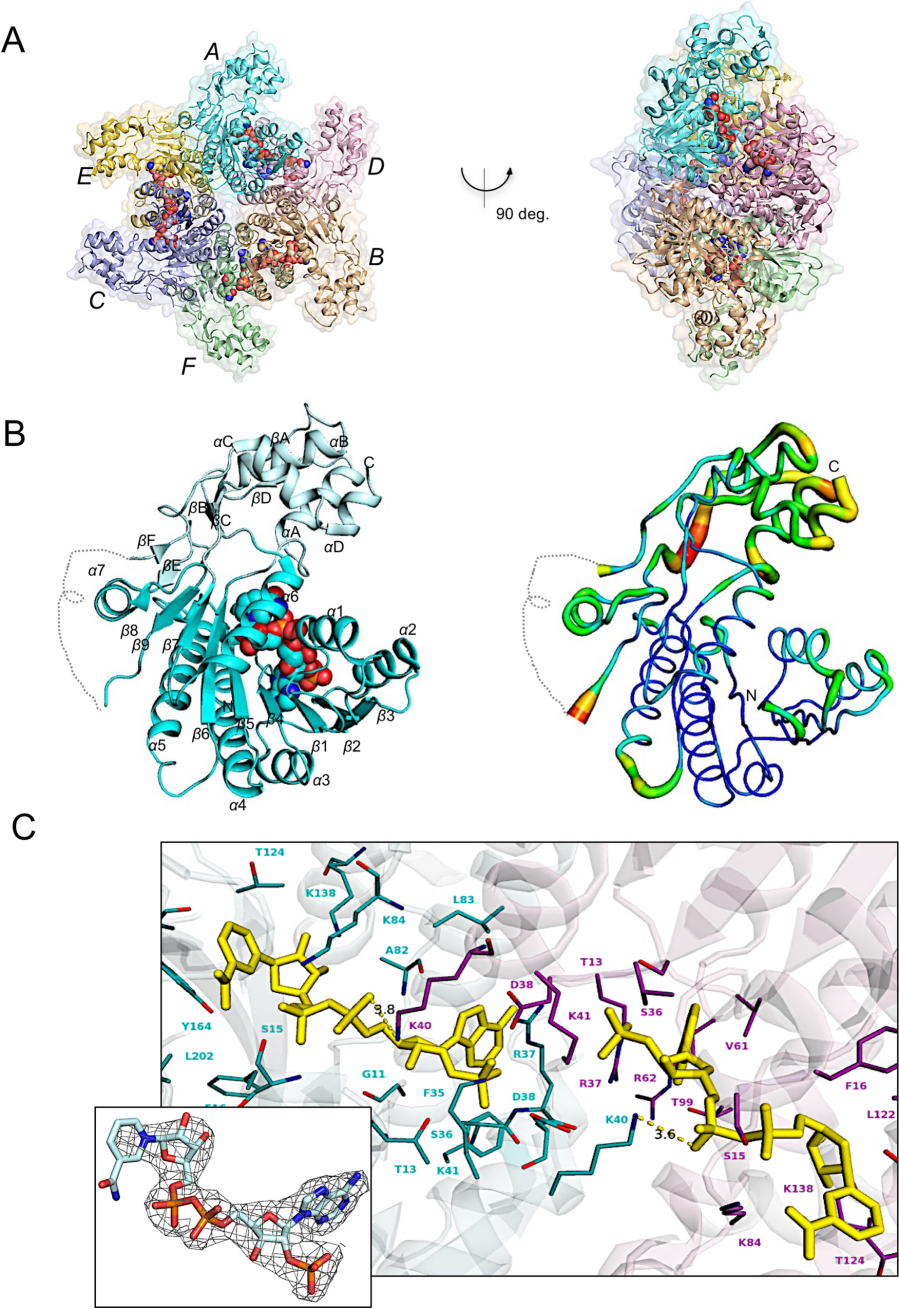
Crystal structure of *Ab-*WbjB (PDB 4j2o) at 2.65Å. (A) Hexamer structure incorporates a trimer of dimers: A-E (cyan, yellow), B-D (orange, pink) and C-F (violet, green). Six bound NADP+ molecules are shown (red) as CPK spheres. (B) Magnification of chain A (left) shows its two-domain architecture: N-terminal Rossmann (cyan) and C-terminal (pale cyan) domains; (right) B-factor putty representation from high (red) to low (blue) values. Polypeptide backbone for which density is absent is dashed. (C) Coenzyme NADP+ (yellow) and surrounding side-chains (chain A, cyan; chain D, pink) at the A-D interface within the *Ab-*WbjB hexamer. Interatomic distances compatible with hydrogen bonding of NADP+ to K40 sidechain is shown (dotted yellow). Inset shows the 2F_o_-F_c_ SA-omit map (grey mesh) contoured at 2.0σ for coenzyme NADP+ (chain A).

Our crystal structure of *Ab*-WbjB solved at 2.65 Å resolution is illustrated in Fig 2. Diffraction datasets of *Ab*-WbjB crystals scaled and integrated in the space group P2_1_2_1_2_1_, (unit cell a = 107.71, b = 114.21, c = 215.04). The crystalline packing showed a hexameric organization in 32 symmetry, i.e. organised as a trimer of paired chains (A-E, B-D and C-F). Six chains of the hexamer overlay with an average root-mean-square deviation (r.m.s.d) of 0.62 Å. Density attributable to coenzyme NADP+ is observed in each of the six protomers, despite no exogenous NADP+ being added during sample preparation or crystallization. No density attributable to substrate could be identified in our structure solution.

The *Ab*-WbjB protomer contains two well-defined domains: the largest encompasses a Rossmann fold, comprising residues 1–165, 200–224, 266–286, and a bound NADP+ molecule. The nine-stranded β-sheet is predominantly parallel (order: 3-2-1-4-5-6-7-9-8) and is flanked by seven α-helices. The smaller C-terminal domain is largely helical (helices αA-αD), but additionally incorporates two discrete β-regions, made up of short parallel (βA, βD) and anti-parallel (βB, βC) strand pairs. Linking segments between the two domains include two short antiparallel β-strands (βE, residues 263–265; βF, 307–309). Relatively high B-factors are observed across the C-terminal domain and its inter-connecting linkages, with density absent for a twenty-residue segment (Fig 2B).

A combination of highly conserved side-chain, backbone, and water-mediated contacts engages coenzyme NADP+ within the N-terminal domain of *Ab*-WbjB (depicted in Fig 2C). This binding site encompasses an elongated hydrophilic cavity formed by loops of the Rossmann fold (β1–α1, β2–α2 and β3–α3) which enclose the adenosine portion of the coenzyme. Several hydrogen bonds play a role in docking the adenine ring (engaging side-chains D60, T99 and V61), and residues R37 and K41 contact the 2’-phosphate of the adenosine ribose. The nicotinamide moiety of NADP+ is projected into the inter-domain region of the fold, allowing both ribose -OH substituents to hydrogen bond with K138, and the amide nitrogen to engage the Y164 carbonyl oxygen.

Importantly, examination of the *Ab*-WbjB hexamer shows the NADP+ associated with chain A to additionally interact with the K40 side-chain emerging into the binding pocket from chain D, i.e. from the adjoining dimer (B-D, see Fig 2C). This facilitates a hydrogen bond from the NADP+ α-phosphate to the lysine quaternary amine. A reciprocal interaction via K40 of chain A stabilises the coenzyme bound in chain D. With six coenzyme molecules bound across the hexamer, these hydrogen-bond pairings recur across all remaining protomeric interfaces, so linking chain B and chain F, and chain C with chain E. Given these interactions act to tether non-dimeric protomer units, binding of coenzyme NADP+ can be seen as integral to locking-in the hexameric organization of *Ab*-WbjB.

### Structural homologs of *Ab-*WbjB

**Fig 3:**
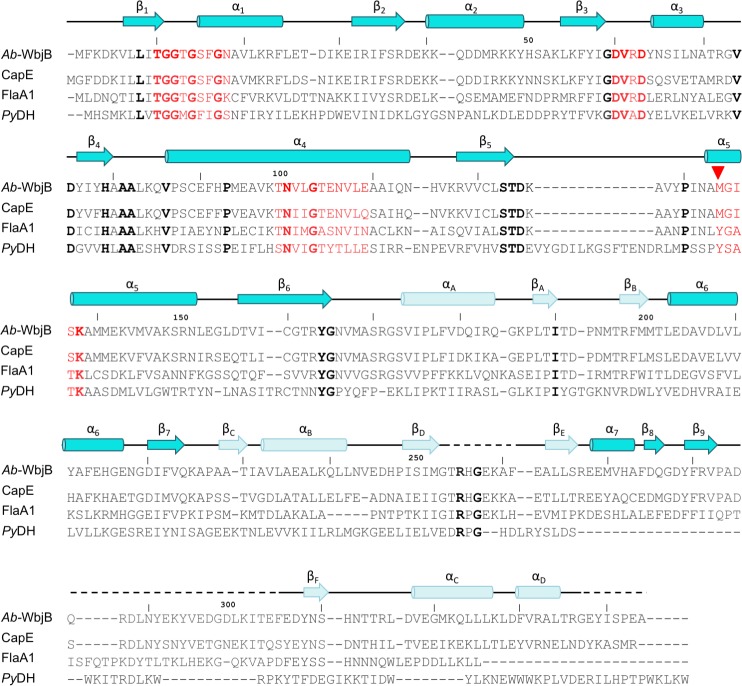
Sequence alignment of *Ab-*WbjB and its structural relatives. Sequences of structural homologs of *Ab-*WbjB (r.m.s.d <3.0 Å); *S*. *aureus* CapE, *H*. *pylori* FlaA1, and *Pyrococcus horikoshii* dehydratase *Py*DH, are shown. Strictly conserved residues (bold) and dinucleotide binding/active site motifs characteristic of SDR enzymes (red) are emphasised. Residue M134, replacing the usual catalytic tyrosine is indicated (▽). Sequences are aligned in T-Coffee [50]. Secondary structure elements of *Ab-*WbjB crystal structure are overlaid to outline N-terminal Rossmann (dark) and C-terminal (light) domains.

Searches for three-dimensional structural homologs (S2 Table) reveal *Ab-*WbjB to be a member of the short-chain dehydrogenase-reductase (SDR) superfamily. *Ab-*WbjB conforms to the ‘extended’ class of SDR enzymes, displaying an embellished C-terminal domain and several signature sequences associated with this particular sub-group [46, 47]. Fig 3 shows the sequence alignment for the closest structural relatives of *Ab-*WbjB (< 3.0 Å r.m.s.d), which share moderate (< 60%) sequence identity. Sequences taken as characteristic of dinucleotide binding [46] are evident: the gly-rich consensus motif at T10 (T-G-x_2_-G-Φ-Ω-G, Φ = hydrophobic, Ω = aromatic), a charged loop, here connecting β3–α3 (D60 –D63), and elements of helix α4 (constituting signature motif Φ-N-Φ_2_-G-T-x_2_-Φ_2_-c, c = charged residue). Immediately obvious from this sequence alignment is disruption at helix α5 of the canonical Y-x_3_-K motif known to form the active site of SDR enzymes [48, 49]. In *Ab-*WbjB, M134 replaces the usual catalytic tyrosine sidechain.

The closest structural homolog to *Ab-*WbjB (r.m.s.d 1.0 Å) is the enzyme CapE from *S*. *aureus* [51]. This bi-functional enzyme catalyzes the dehydration and epimerization of UDP–sugars for the synthesis of CPS [43]. Three states of CapE have been defined through crystallography [51, 52]: an apo form with substrate analogue bound (PDB 3w1v), and two holo forms with NADP+ bound (one with no substrate, PDB 3vvb; the other with by-product UDP-xylo-sugar in the active site, PDB 4g5h). A second close structural homolog (r.m.s.d 1.8 Å) is FlaA1 from *H*. *pylori* [31], also involved in saccharide biosynthesis [53]. Crystal structures of FlaA1 have been solved with substrate UDP-GlcNAc in place, as well as bound to various substrate analogs (UDP, UDP-glucose (UDP-Glc), and UDP-galactose (UDP-Gal)) [31]. These FlaA1 structures all incorporate coenzyme NADP+.

**Fig 4:**
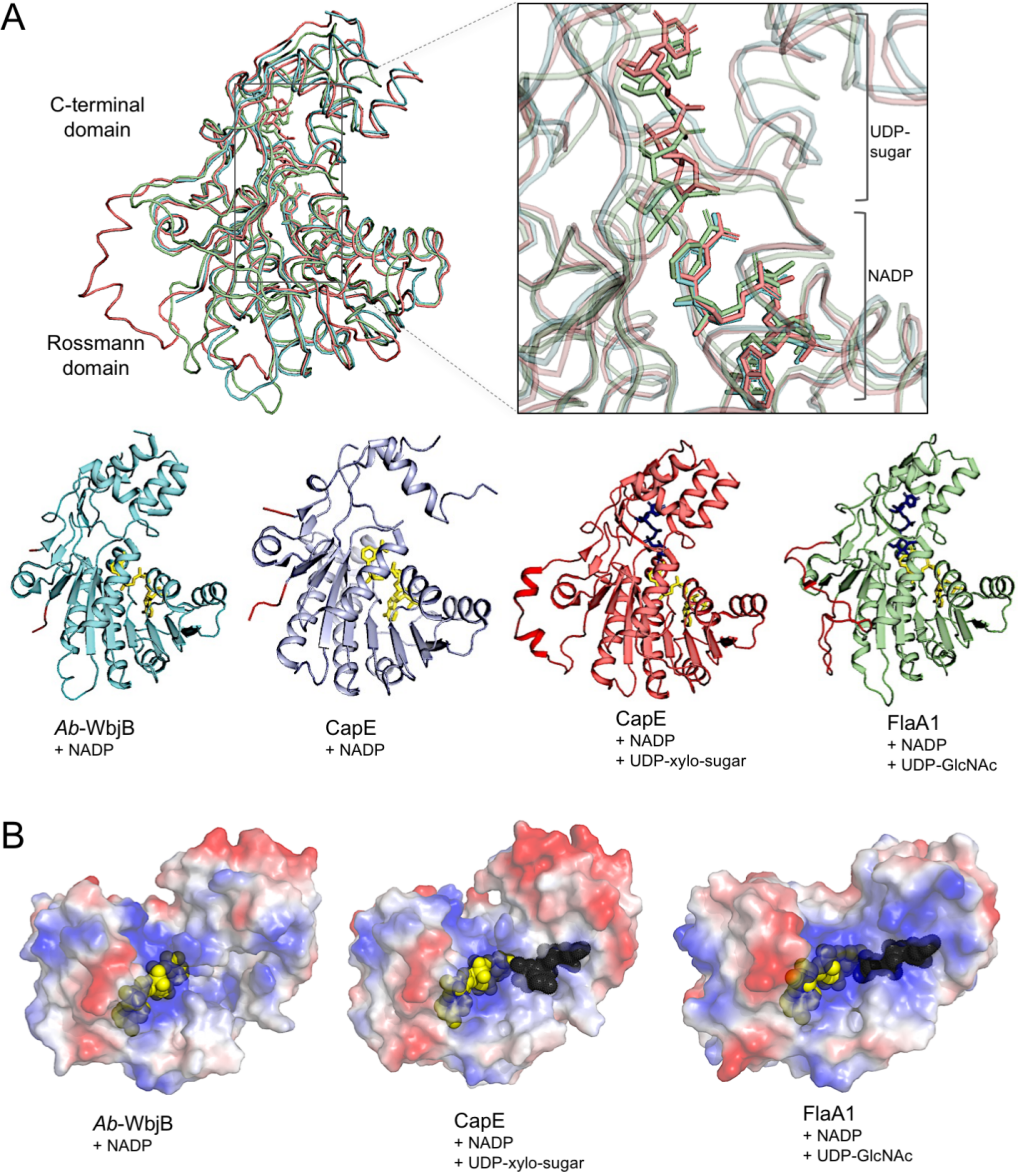
Structural comparison of *Ab-*WbjB with CapE and FlaA1. (A) Backbone Cα trace of *Ab-*WbjB (PDB 4j2o, cyan) overlaid with those of holo CapE + UDP-xylo-sugar (PDB 4g5h, pink) and holo FlaA1 + UDP-GlcNAc (PDB 2gn4, green). Magnification (right) depicts coenzyme NADP+ and substrate/substrate analogues (sticks) bound to respective structures. Cartoons of the various SDR structures (bottom) are shown with coenzyme (yellow sticks) and substrate/substrate analogue (blue sticks). Backbone for polypeptide segment connecting strands β9 - βF is highlighted (red) in each case to emphasize the variable region (B) Surface representation of *Ab-*WbjB, CapE (PDB 4g5h) and FlaA1 (PDB 2gn4) coloured according to residue charge (generated in PyMOL [39]).

The structural conservation between *Ab-*WbjB, CapE and FlaA1 is illustrated in Fig 4. The overlay shows a close fit for both Rossmann and C-terminal domains, as well as of their relative dispositions. While there is no substrate bound in our *Ab-*WbjB crystal structure, a substrate is clearly evident within the C-terminal domains of its close relatives. Our crystal structure of *Ab-*WbjB contains a highly charged cavity immediately adjacent to the NADP+-binding site (see Fig 4B). This cleft is lined with several basic side-chains (R171, K126 and K138) from the β6-αA loop and the highly flexible helix αA of the C-terminal domain, as well as acidic (D125) and polar (S170 and S173) groups. In each of the homologous enzymes CapE and FlaA1, a similarly located electropositive feature defines the substrate-binding pocket [31, 51, 52]. Thus, by analogy, we insinuate a similar chemistry for *Ab-*WbjB, and propose this to be a suitable site for binding of UDP-linked hexose substrate.

The most marked variation across these SDR relatives is seen for a long segment (26 residues) connecting strands β9 to βF, (coloured red in Fig 4A). In our *Ab-*WbjB holo structure (NADP+ bound, no substrate), this constitutes a dynamic element with no density evident for residues R287—E306. The corresponding region of CapE when substrate-free (the holo structure) also displays dynamic disorder within the crystal form. With substrate analogue bound, however, this region of CapE becomes ordered with the β9-βF connecting segment seen to include two short α-helices. This structural element extends well away from the two core domains and has been defined as a third “latch” domain for CapE [51, 52]. In contrast, the tertiary structure of FlaA1 bound with UDP-GlcNAc has the corresponding segment stabilised within an extended β-sheet structure, incorporated as a new β-strand (β10) within the central sheet of the Rossmann domain (see Fig 4). Thus, on binding to substrate, it is likely that the β9-βF loop of *Ab-*WbjB will become reorganised and take on a more highly-structured form.

### Hexameric organization of *Ab*-WbjB

As depicted in our crystal structure, *Ab-*WbjB assembles into a hexamer, a quaternary form also previously reported for enzymes CapE and FlaA1 [31, 51]. This oligomeric organization is rare amongst the SDR family, which more characteristically tend to dimeric or tetrameric forms [46, 54]. Analysis by SEC on our *Ab-*WbjB samples (see S1 Fig) indicates a hydrodynamic radius consistent with ~220 kDa native mass, verifying a hexameric solution state for this 41 kDa enzyme.

The hexameric organisation of *Ab-*WbjB incorporates 32-point group symmetry in which each protomer engages two major interfaces (depicted in Fig 2). Evaluation of intermolecular contacts within each dimer component (A-E, B-D or C-F) indicates an internal interface of ~960 Å^2^. This interface principally engages helices α4 and α5 from each component chain as a four-helical bundle subassembly, a motif responsible for the dimeric quaternary state generally characteristic of SDR enzymes [54].

A second interface (measured as ~970 Å^2^) engages chain A with chain D (and, by symmetry, pairs chains B-F and C-E). As discussed above, this contact is essential for the hexameric grouping of chains, and incorporates both protein-protein as well as specific protein-coenzyme interactions (Fig 2C). This intricate association of chains includes two salt bridges (between D38—K41 and D63—K98 side-chains), as well as polar and hydrophobic interactions. This interface within the *Ab-*WbjB hexamer is relatively rare within the SDR family, but has also been observed in CapE and FlaA1 structures. We note that residues contributing to salt bridges (D38, K41, D63, K98 side-chains) are fully conserved in CapE and FlaA1 sequences (Fig 3).

For some of our laboratory samples of *Ab-*WbjB, a dimer form was occasionally observed in solution (an example is presented in S1 Fig). We presume this to have occurred where lower amounts of coenzyme NADP+ were scavenged by the protein during its recombinant preparation. We note similar reports for solutions of FlaA1, occasionally observed as dimeric [55] or tetrameric [31] populations. A similar phenomenon has previously been described for human UDP-Glucose 6-dehydrogenase (hUGDH) [56], a member of the long-chain dehydrogenase/reductase family, sometimes seen as mixed dimeric/tetrameric/hexameric populations [56, 57]. The stabilisation of catalytically-active hexameric hUGDH in solution was attributed to a cofactor-sensitive allosteric switch [56]. We postulate that the coenzyme interactions at the chain A—chain D interface in *Ab-*WbjB are also likely to act as an analogous molecular switch to regulate the quaternary organisation of this SDR enzyme. A similar mechanism may also occur for its relatives CapE and FlaA1.

### Insights into the active site of *Ab-*WbjB

As no substrate was captured during crystallisation of *Ab*-WbjB, a screening for potential substrate analogs was conducted. This involved a search for potential binding partners amongst a library of ~1100 compounds, which included typical bioactive ligands and cofactors, biochemical pathway intermediates, nucleotides, carbohydrates, salts and metals. Evidence for binding was detected by thermostability measurements. In the presence of excess NADP+ (to 10 mM) and no potential substrate, recorded T_m_ values were increased by 3–5°C, indicating improved stability for *Ab*-WbjB when bound with its coenzyme. Elevation of T_m_ only occurred for cocktails which included UDP-GlcNAc (80°C), and GalNAc (78°C). The specificity of this interaction is indicated by the fact that 81 of 96 cocktail conditions (with NADP+ present) did not alter the protein stability (T_m_ = 74°C).

**Fig 5:**
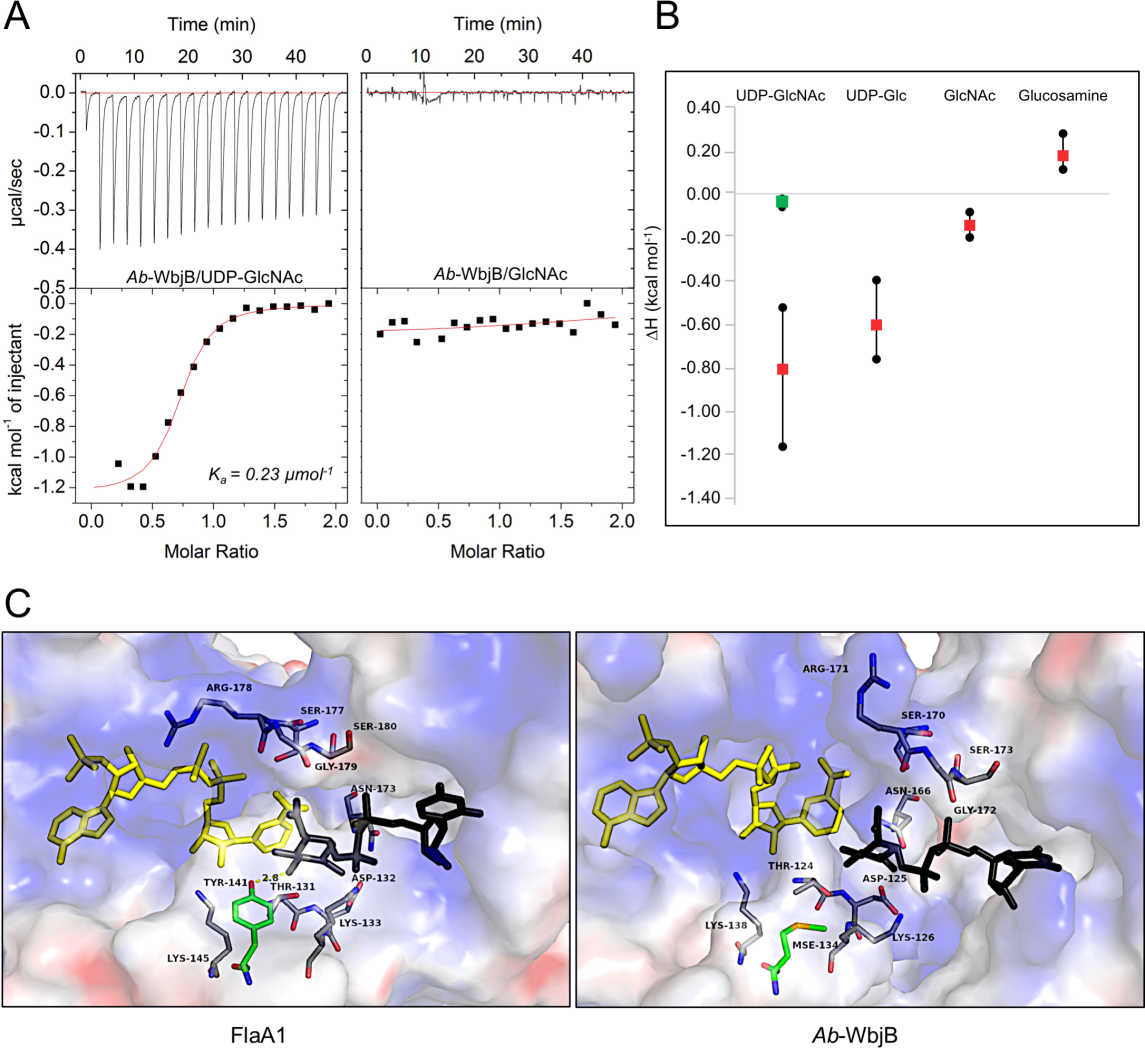
Definition of *Ab*-WbjB substrate chemistry. (A) ITC response for titration 25°C of *Ab*-WbjB (100 μM) with UDP-GlcNAc (1 mM) and GlcNAc (1 mM). All titrations were carried out in HEPES buffer (50mM, pH 7.5) with 200 mM NaCl. Curve of best fit is drawn by Origin 7 software. (B) Binding enthalpies for *Ab-*WbjB (red) and M134A-WbjB (green) with specified hexose sugars. Enthalpy values derived across triplicate ITC measurements are indicated. (C) Active site with coenzyme for (left) FlaA1 [31] and (right) *Ab*-WbjB. In both cases, substrate UDP-GlcNAc is shown (black) as observed in FlaA1(PDB 2gn4) and modelled in *Ab*-WbjB from coordinates of PDB 2gn4. Side-chains conserved in both enzymes are shown (grey), as well as tyrosine of the canonical Y-x_3_-K motif (green).

The binding of specific hexose sugars to *Ab-*WbjB prepared in presence of a reductant (TCEP) was further monitored by ITC. Individual titrations were performed with UDP-GlcNAc, as well as UDP-Glc, GlcNAc, and glucosamine. As depicted in Fig 5, the association of UDP-GlcNAc with *Ab*-WbjB indicates relatively moderate binding (K_a_ = 0.23 μmol^-1^) in an exothermic process (ΔH = -0.8 kcal mol^-1^). Our calorimetric experiments in the presence of UDP-Glc also showed binding, but at lower affinity (ΔH = -0.6 kcal mol^-1^), demonstrating preference for the N-acetylated derivative. No clear binding was indicated to GlcNAc and glucosamine, implying the need for substrate with dinucleotide structure. These binding data confirm UDP-GlcNAc to be a valid substrate for *Ab*-WbjB, corroborating inference made from our structural analysis (discussed above).

Immediately evident within the structure of *Ab*-WbjB is the conservation of chemical features proposed to form the substrate-binding pocket of SDR enzymes (see Fig 4). Of its homologs, only the crystal structure of FlaA1 includes both coenzyme (NADP+) and substrate (UDP-GlcNAc) molecules [31]. As illustrated in Fig 4A, the substrate hexose ring in the structure of FlaA1 shows a catalytic conformation with respect to coenzyme, not seen in the crystal structures of CapE bound only with substrate analogues [51, 52]. To better understand the active site chemistry of *Ab*-WbjB, coordinates for UDP-GlcNAc were superimposed from the FlaA1 crystal structure (PDB 2gn4). Fig 5 highlights side-chains located near substrate in FlaA1, and compares the corresponding chemical environment in *Ab*-WbjB around a modelled substrate.

For the sequential steps of oxidation, dehydration and epimerisation of UDP-GlcNAc carried out by FlaA1, side-chains involved in the catalytic mechanism include D132, K133 and Y141 [31]. In *Ab-*WbjB, two of these side-chains are preserved, namely D125 and K126 (see Fig 5). However, no tyrosine group occupies the required active site location in *Ab*-WbjB (Y141 in FlaA1); notably this position is instead occupied by a methionine (M134) side-chain. (In the instance of this crystal structure, this residue occurs as selenium-derivative). To probe the impact of M134 inserted within the canonical Y-x_3_-K active site motif, a single-site mutant M134A-WbjB was prepared. When binding of UDP-GlcNAc was measured by ITC for this mutant, a ΔH value of -0.07 kcal mol^-1^ was derived, i.e. considerably reduced relative to the Met-containing native protein. Therefore, loss of steric bulk (and, possibly, nucleophilicity) at this location has compromised the substrate-binding capabilities of *Ab*-WbjB. Attempts to isolate the variant M134Y unfortunately proved unsuccessful, likely due to misfolded product.

In FlaA1, the hydroxyl substituent of Y141 is proposed to act as a base to initiate formation of the UDP-linked 4-keto sugar intermediate [31]. On consideration of the three-dimensional arrangement of side-chains at the substrate-coenzyme site, we propose that *Ab*-WbjB instead has the potential to engage the nearby side-chain T124 to accomplish this step. As depicted in Fig 5, the–OH group of this residue is positioned appropriately, located between M134 and the C4 hydroxyl of (modelled) substrate. Remaining catalytic steps of dehydration and reduction of substrate would likely engage side-chains of K126 and D125, following the mechanism previously described for FlaA1 [22, 31]. We note a similar arrangement for the active site of *S*. *aureus* CapE: the tyrosine of the Y-x_3_-K sequence motif is also replaced by a methionine, with a threonine sidechain (located C-terminal to strand β5) appropriately sited for catalysis.

## Conclusion

We have here utilised crystallography to confirm the annotated function of the polysaccharide biosynthesis cluster, RGP01, from a community-derived strain of *A*. *baumannii*. Our crystal structure of *Ab*-WbjB suggests it to be an epimerase and a member of the extended SDR family, related to homologs in *H*. *pylori* and *S*. *aureus*. We have demonstrated binding to UDP-GlcNAc, indicating *Ab*-WbjB likely to be responsible for the conversion of this substrate to UDP-L-FucNAc as a biosynthetic precursor step in surface glycoside formation.

*Ab*-WbjB forms a hexameric organisation rarely depicted in structures of SDR enzymes. We propose the coenzyme NADP+ to contribute to an allosteric switch, regulating the formation of a hexamer and thereby the activity of *Ab-*WbjB. Furthermore, the catalytic site seen in *Ab*-WbjB constitutes sidechains contributed from protein locations beyond the canonical Y-x_3_-K sequence motif of the SDR enzyme family. The location of the–OH substituent of T124 is suggestive of its capacity to act as an alternate base in this enzyme; we note a similar distinctive site for the CapE enzyme of *S*. *aureus* [58]. Thus, with the benefit of three-dimensional characterisation, we infer side-chains N-terminal to the SDR consensus catalytic motif [46] might serve as alternate participants in active site chemistry of these epimerases.

Surface polysaccharides such as CPS are important outer membrane components in Gram-negative bacteria, responsible for molecular processes intrinsic to host recognition and defence, and contributing to pathogen virulency. Across various *Acinetobacter* spp., RGPs associated with CPS biosynthesis show great diversity [17], thereby promoting structural heterogeneity of CPS at the molecular level [21]. A greater understanding of the functional specificity of enzymes encoded within these clusters clarifies biosynthetic routes for functionalised hexose sugars and their subsequent glycodiversification for CPS biosynthesis. Our work contributes an additional structure to sugar-modifying enzymes solved to date, extending knowledge of sequence variants of the SDR family. Mechanistic understanding of specific enzymes responsible for generating core sugars of surface glycostructures in *A*. *baumannii*, such as *Ab*-WbjB, can be expected to contribute to future immunogenic treatments for infection control.

## Supporting information

S1 Fig Oligomeric analysis of *Ab-*WbjB. (A) SDS-PAGE of purified *Ab*-WbjB protein sample. Gel is stained with Coomassie Brilliant Blue (B) SEC profile in HEPES buffer (pH7.5, with 200mM NaCl, 5% glycerol)) on Superdex 200 for *Ab*-WbjB. The elution of calibration standards is indicated (red).
(TIF)

S1 Table *Ab-*WbjB mutants prepared by site-directed mutagenesis. (PDF)

S2 Table Structural homologs of *Ab-*WbjB. (PDF)
