# Supplementary figures and images for: Crystal structure of a UDP-GlcNAc epimerase for surface polysaccharide biosynthesis in Acinetobacter baumannii
Source: PLoS One. 2018 Jan 19;13(1):e0191610. doi: 10.1371/journal.pone.0191610 (PMC5774825; doi:10.1371/journal.pone.0191610)

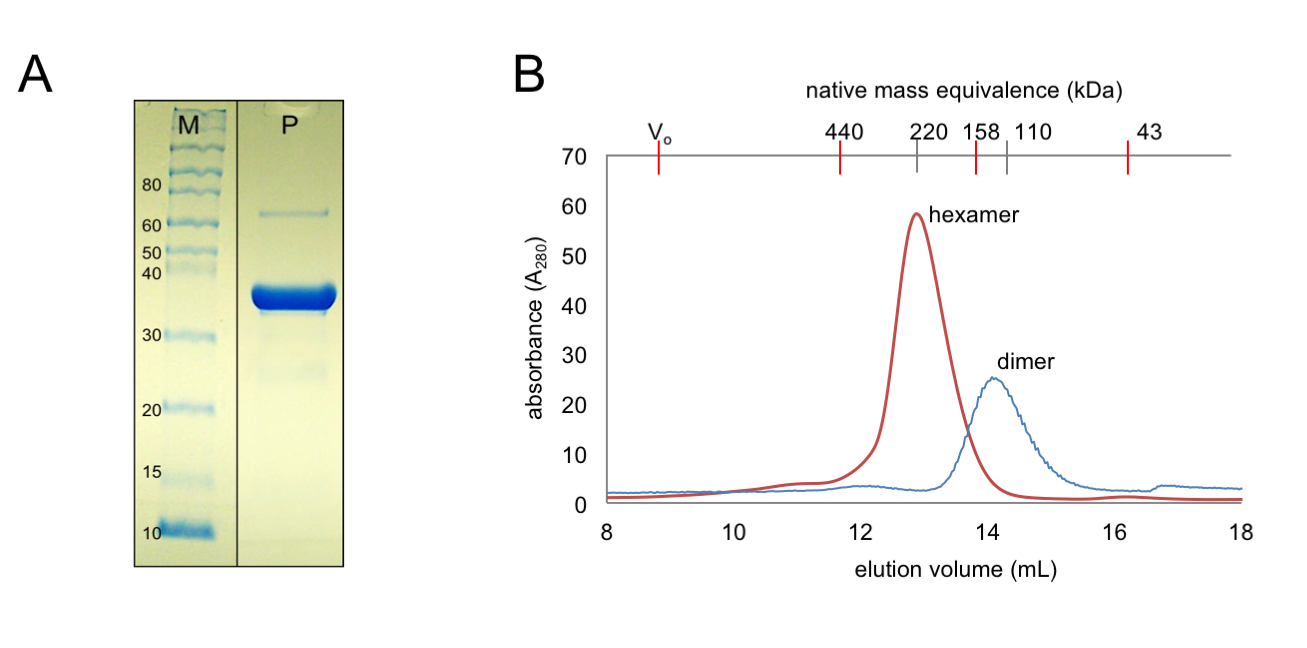

Supplement: S1 Fig — (A) SDS-PAGE of purified Ab-WbjB protein sample. Gel is stained with Coomassie Brilliant Blue (B) SEC profile in HEPES buffer (pH7.5, with 200mM NaCl, 5% glycerol)) on Superdex 200 for Ab-WbjB. The elution of calibration standards is indicated (red). (TIF) [file pone.0191610.s001.tif]
