# Supplementary material for: Crystal structure of a UDP-GlcNAc epimerase for surface polysaccharide biosynthesis in Acinetobacter baumannii
Source: PLoS One. 2018 Jan 19;13(1):e0191610. doi: 10.1371/journal.pone.0191610 (PMC5774825; doi:10.1371/journal.pone.0191610)
Supplement: S1 Table — (PDF) [file pone.0191610.s002.pdf]

S1 Table. ***Ab*****-WbjB mutants prepared by site-directed mutagenesis**

Mutant	Primer sequence (5'-3')
M134A	F - GCAGTTTACCCAATTAATGCAGCGGGTATTTCTAAAGCCATGATGG
	R - CCATCATGGCTTTAGAAATACCCGCTGCATTAATTGGGTAAACTGC
M134Y	F - GCAGTTTACCCAATTAATGCATACGGTATTTCTAAAGCCATGATGG
	R - CCATCATGGCTTTAGAAATACCGTATGCATTAATTGGGTAAACTGC
M134L	F - GCAGTTTACCCAATTAATGCACTCGGTATTTCTAAAGCCATGATGG
	R -CCATCATGGCTTTAGAAATACCGAGTGCATTAATTGGGTAAACTGC
