# Supplementary material for: Crystal structure of a UDP-GlcNAc epimerase for surface polysaccharide biosynthesis in Acinetobacter baumannii
Source: PLoS One. 2018 Jan 19;13(1):e0191610. doi: 10.1371/journal.pone.0191610 (PMC5774825; doi:10.1371/journal.pone.0191610)
Supplement: S2 Table — (PDF) [file pone.0191610.s003.pdf]

S2 Table. Structural homologues of *Ab*-WbjB

PDB file	Dali Z-score[a]	r.m.s.d (Å)[b]	sequence identity (%)	enzyme name	source organism	quaternary structure	Coenzyme/ substrate
3vvb	39.4	1.0	68	CapE	*S. aureus*	hexamer	NADP
2gn4	38.1	2.1	39	UDP-GlcNAc-8-dehydratase/ FlaA1	*H. pylori*	hexamer	NADP
2hun	27.0	2.3	23	DTDP-glucose 4,6-dehydratase	*P. horikoshii*	dimer	NAD
1wvg	26.1	2.8	20	CDP-glucose 4,6-dehydratase	*S. typhi*	tetramer	APR/CXY
1rkx	25.6	2.7	22	CDP-glucose 4,6-dehydratase	*Y. pseudotuberculosis*	tetramer	NAD
3lu1	25.2	2.8	22	WbgU	*P. shigelloides*	dimer	NAD/ UDP-GalNAc
2pzm	24.9	2.3	21	putative nucleotide sugar epimerase/ dehydratase	*B. bronchiseptica*	dimer	NAD/ UDP
1g1a	24.3	2.8	23	DTDP-glucose 4,6-dehydratase	*S. enterica*	dimer	NAD/ sulphate ion
3ehe	22.9	2.9	23	UDP-glucose 4-epimerase	*A. fulgidus*	dimer	NAD

[a] Statistical significance of best alignment as calculated by DALI. This score includes weighting according to sequence identity levels.
[b] Position deviation over main-chain Cα atoms as calculated within DALI
